# Editorial: Current Challenges in Cardioembolic Stroke

**DOI:** 10.3389/fneur.2021.688371

**Published:** 2021-04-27

**Authors:** Blanca Fuentes, George Ntaios, Jukka Putaala

**Affiliations:** ^1^Department of Neurology and Stroke Center, La Paz University Hospital and Universidad Autónoma de Madrid, Madrid, Spain; ^2^Department of Internal Medicine, Faculty of Medicine, School of Health Sciences, University of Thessaly, Larissa, Greece; ^3^Department of Neurology, Helsinki University Hospital and University of Helsinki, Helsinki, Finland

**Keywords:** stroke, cardioembolic, editorial, research, cryptogenic stroke

Cardioembolism is one of the most frequent causes of ischemic stroke, accounting for at least 20% of all ischemic strokes. Moreover, cardioembolic stroke entails higher severity and poorer outcome compared to other etiologies. Atrial fibrillation is the most frequent underlying disease, although several other cardiac disorders such as ventricular akinesia, valvular heart disease, acute myocardial infarction, endocarditis, monogenic diseases with cardiac involvement, or complicated interventional cardiac procedures can also be involved in the development of cardioembolic stroke. Direct oral anticoagulants have revolutionized stroke prevention in patients suffering from atrial fibrillation, however, more knowledge is needed regarding the long-term outcomes in patients treated with these drugs.

In recent years, much attention has been paid to the search for hidden cardioembolic sources for ischemic stroke, and the “embolic stroke of unknown source” (ESUS) concept was raised to improve the detection of occult embolic diseases, mainly paroxysmal atrial fibrillation. However, the failure of direct oral anticoagulants in recent clinical trials with patients diagnosed with ESUS has raised concerns regarding the appropriateness of a low-intensity diagnostic workup and points to the return of the diagnostic concept of cryptogenic stroke. This highlights the need to increase the search for potential hidden etiologies through advanced diagnostic procedures.

This Research Topic issue provides an up-to-date collection of manuscripts focused on the challenge of diagnosis and management of cardioembolic strokes.

The role of echocardiography is reviewed by Pagola et al. highlighting the potential application of Point of Care UltraSound (POCUS) as a helpful screening method at the Stroke Unit. Furthermore, Arnold et al. provide a comprehensive review on the most promising biomarkers for the diagnosis of cardioembolic stroke, including blood-based, genomic, transcriptomic, metabolic, electrocardiographical, echocardiographical, and advance cardiac imaging biomarkers. They discuss the existing evidence for possible clinical applications in risk stratification and in accelerating etiological classification as well as optimal management, from a perspective of the forthcoming implementation of personalized medicine in stroke management.

The importance of atherosclerotic plaques in the aortic arch as a potential cause of embolic stroke has been underestimated in the majority of the etiological classifications of stroke, being relegated in most of them to stroke of undetermined origin, mainly because of a clear recommendation for its systematic search in the stroke patients has not been established. The narrative review by Viedma-Guiard et al. provides an excellent and up-to-date overview of the role of aortic sources of embolism in ischemic stroke, focusing on the diagnostic challenges and the current evidence for the optimal treatment in stroke prevention.

Recently the ESUS concept has been called into question. This concept was proposed in 2014 by the Cryptogenic Stroke/ESUS International Working Group under the rationale of the common underlying thromboembolism mechanism in the group of patients with non-lacunar ischemic strokes in which the basic diagnostic approach did not found any undoubtable cause for the stroke. This concept included minor-risk or covert cardiac sources (such as covert paroxysmal atrial fibrillation), paradoxical embolism and non-occlusive atherosclerotic plaques in the aortic arch, cervical or intracranial arteries (1). However, as long as this concept was implemented in clinical practice, it has been clear that the majority of stroke patients classified as ESUS are more similar to atherosclerotic stroke patients in their clinical features and that non-occlusive atherosclerotic plaques might be the most common source of embolism in those cases. The manuscript by Wan et al. also provides new data on this atherosclerotic-lookalike clinical profile in a prospectively collected series of 119 acute ischemic stroke patients. To deepen the current controversy surrounding the ESUS concept, Fuentes et al. list several arguments against the use of the ESUS concept in clinical practice, advising stroke physicians to be smart in the search for underlying causes of ischemic stroke, optimizing advanced diagnostic procedures according to the patient's and stroke's characteristics, attempting to find the correct diagnosis for stroke patients and reducing rates of cryptogenic stroke diagnosis.

In the field of stroke prevention in cardioembolic strokes, this Research Topic issue includes two interesting manuscripts. The prospective multicentre RESTAIC Registry by Gutiérrez-Zúñiga et al. explored the differences in ischemic and haemorrhagic events when using oral anticoagulation for secondary stroke prevention according to the type of anticoagulant treatment (vitamin K antagonists or direct oral anticoagulant drugs) showing a low frequency of bleeding complications, with no significant differences between anticoagulant drugs at long-term follow-up (of up to 3 years from a stroke). Besides, they provide new insights on the main reasons for switching oral anticoagulant drugs in clinical practice, being the majority of them due to stroke recurrences and labile INR. The other manuscript on stroke prevention is the protocol for LASER (Lixiana Acute Stroke Evaluation Registry) a randomized clinical trial and an associated registry of early anticoagulation with edoxaban after ischemic stroke in patients with atrial fibrillation. It is aimed at recruiting 150 patients with ischemic stroke and atrial fibrillation who will be randomized within 5 days of stroke onset to early or delayed edoxaban initiation (Alrohimi et al.).

Finally, this issue also includes a case report by Sarti et al. highlighting the relevance of atrial appendage morphology on patients with atrial fibrillation and resistant stroke despite adequate anticoagulant therapy.

We would like to thank all the contributing authors for their manuscripts in this challenging time in which the COVID-19 pandemic has severely impacted stroke care and research, limiting the number of the expected manuscripts for this Research Topic issue. We hope that the pandemic will soon disappear, and we can refocus all our efforts on fighting stroke, one of the leading causes of death and the first cause of disability worldwide.

## Author Contributions

All authors contributed by drafting the work or revising it critically for important intellectual content and all of them provided approval for publication of the content.

## Conflict of Interest

BF: Advisory Board (Bayer); Spearker's honoraria (BMS-Pfizer, Daichi-Sankyo); Travel grants to attend scientific meetings (Daichi-Sankyo, BMS-Pfizer), Participation in clinical trials (RESPECT-ESUS, NAVIGATE-ESUS). GN: Speaker fees/Advisory Boards/Research support from Amgen; Bayer; BMS/Pfizer; Boehringer-Ingelheim; Elpen; European Union; Galenica; Sanofi; Winmedica. JP: Research funding: Helsinki and Uusimaa Hospital District, Academy of Finland, Business Finland, St Jude Medical, Pfizer. Research collaboration: Nokia Technologies, Bittium, BcB Medical, Vital Signum; Speaker's honorary: Boehringer-Ingelheim, BMS-Pfizer, Bayer, Abbot; Advisory Board: BMS-Pfizer, Boehringer-Ingelheim, Bayer, MSD, Portola. Other: ESO guideline working group: Post-stroke hyperglycemia, secondary prevention in patients with AF. Finnish Duodecim guideline working group: ischemic stroke and TIA; Terve Media: Visiting editor.
